# First Report of the Larch Longhorn (*Tetropium gabrieli* Weise, Coleoptera: Cerambycidae: Spondylidinae) on *Larix* spp. in Lithuania

**DOI:** 10.3390/insects12100911

**Published:** 2021-10-06

**Authors:** Jūratė Lynikienė, Vytautas Tamutis, Artūras Gedminas, Adas Marčiulynas, Audrius Menkis

**Affiliations:** 1 Institute of Forestry, Lithuanian Research Centre for Agriculture and Forestry, Liepu Str. 1, Girionys, LT-53101 Kaunas District, Lithuania; arturas.gedminas@lammc.lt (A.G.); adas.marciulynas@lammc.lt (A.M.); 2 Kaunas Botanical Garden, Vytautas Magnus University, Ž. E. Žilibero St. 6, LT-46324 Kaunas, Lithuania; dromius@yahoo.com; 3 Kaunas T. Ivanauskas Zoological Museum, Laisvės al. 106, LT-44253 Kaunas, Lithuania; 4 Department of Forest Mycology and Plant Pathology, Swedish University of Agricultural Sciences, Uppsala BioCenter, P.O. Box 7026, SE-75007 Uppsala, Sweden; audrius.menkis@slu.se

**Keywords:** *Larix* spp., forest stands, insect pest, *Tetropium*, larch longhorn

## Abstract

**Simple Summary:**

*Tetropium gabrieli* is a longhorn beetle that usually attacks weakened larch trees. During prolonged summer droughts, *T. gabrieli* can form outbreaks, causing damages to larch stands. Although it is known in several European countries, in Lithuania it was detected for the first time in 2019. The detection of *T. gabrieli* indicates potential secondary damages to European larch and other larch species growing in the area.

**Abstract:**

*Tetropium gabrieli* is known to be native to the Alps in Europe where it breeds in European larch (*Larix decidua*), but it has spread to other areas and was reported in Poland, Sweden, Denmark and Belorussia. Although *T. gabrieli* is considered an important secondary pest of *Larix* spp., it can be particularly harmful to trees subjected to abiotic stress. Here we report that in Lithuania, *T. gabrieli* was for the first time captured in 2019 using sticky traps attached to *Larix* spp. trees. Two adult beetles were trapped at two different sites in central Lithuania, and this was in the period between 10th of May and 5th of June. Regarding potential threats caused by this insect pest, this new finding requires special attention, particularly on its biology, ecology, and local distribution.

## 1. Introduction

The Holarctic genus *Tetropium* Kirby, 1837 (Cerambycidae, Spondylidinae) includes only a limited number of species. Currently, there are 14 species, which are known in the Palaearctic region [1] and among these, 13 are regarded as native to the Western Hemisphere [2]. In Lithuania, there are two reported *Tetropium* species, namely *T. castaneum* (Linnaeus, 1758) and *T. fuscum* (Fabricius, 1778) [3]. Both of these are considered as important secondary pests of *Picea* spp. and *Pinus* spp. trees across their distribution range [4,5,6]. Among the destructive *Tetropium* species, *T. gabrieli* (Weise, 1905) was reported being native to the Alps in central Europe where it breeds in European larch (*Larix decidua* Mill.) [7,8]. In Lithuania, it was not observed previously, but listed in the catalogue of Lithuanian beetles as a probable for the local fauna [9]. In contrast to *T. castaneum* and *T. fuscum*, which are characterized by polyphagia on different coniferous trees, *T. gabrieli* is strictly associated with *Larix* spp. and only occasionally found in other coniferous trees [10,11,12]. Like other *Tetropium* longhorns, *T. gabrieli* is considered to be a secondary pest, breeding mainly in dying or severely stressed standing trees [5], and thus can be more harmful to trees during hot and dry summers [5,8,13]. The planting of *Larix* spp. outside its natural range of distribution has enabled *T. gabrieli* to spread and establish itself in several European countries, including Poland, Sweden, Denmark, and Belorussia [1]. 

In Lithuania, *Larix* spp. was introduced in the early 19th century. However, it should also be considered that in the past, *L. decidua* could grow naturally in the territory of Lithuania [14]. During the last 150–200 years, there were several larch species, including *L. decidua* Mill., *L. polonica* Racib. (Sin. *L. decidua* subsp. *polonica* (Racib.) Domin.), *L. archangelica* Lawson and *L. sibirica* Ledeb., that were planted in Lithuania [15]. Although *Larix* spp. occupy only ca. 900 ha [16], damages caused by insect pests may constitute a significant threat to these stands [17]. Consequently, a new finding of *T. gabrieli* in Lithuania requires special attention, particularly on its biology, ecology, and local distribution.

The aim of this study was to report the first finding of *T. gabrieli* in Lithuania and to discuss its possible habitats, impact, and distribution in the region. 

## 2. Materials and Methods

The species identification of the host trees is problematic due to frequent hybridization [15,18], and thus, is referred to as *Larix* spp. The study was carried out in 2019 and investigated insect communities associated with *Larix* spp. trees (data not shown). The study sites were at ten *Larix* spp. stands, which were selected based on the forest inventory data (Table 1).

At each site, sticky traps, that were placed on stems of five living and five dead *Larix* spp. trees, were used to capture insects [21,22]. Sticky traps were 20 × 20 cm in size, and the polyethylene sheets treated with the non-drying sticky resin (Pestifix, Flora, Tallinn, Estonia). Two traps were attached on the opposite sides of each tree stem at the height of ca. 1.5 m above the ground. These traps were used from the beginning of May until the end of August 2019. During this period, sticky traps with trapped insects were collected once a month, replacing them with new ones, which resulted in three time points (June, July and August). Collected traps were transported on the same day to the laboratory and stored at +5 °C until the analysis and identification of insect species. The prepared specimens were identified according to external characters following the descriptions published by Weise [23], Harde [24], Bíly & Mehl [25]. Length of pronotum, was measured in the midline from the apical margin to the basal margin. Width of pronotum was measured in the distance beside visible lateral margins on its widest place. The morphological assessment was done using stereomicroscope Motic SMZ 168 (Motic Asia, Hong Kong, China). Macrophotographs were taken using a Nikon Z50 camera equipped with a Laowa 25 mm f/2.8 2.5-5X Ultra Macro lens (Canon, Tokyo, Japan).

## 3. Results and Discussion

**Figure 1:**
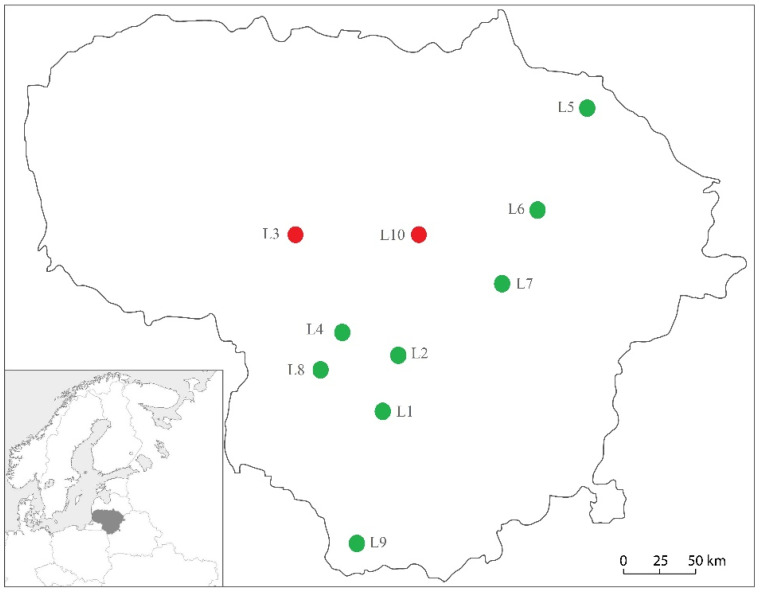
Map of Lithuania showing investigated *Larix* spp. stands (L1–L10): red circles indicate the presence of *T. gabrieli*, and green circles—its absence.

*Tetropium gabrieli* was trapped at two study sites in central Lithuania (Figure 1), i.e., the first specimen at the L3 site and the second at the L10 site. The distance between these sites is ca. 60 km. At the L3 site, *Larix* spp. grows in monocultures on fertile but temporarily waterlogged soil characterized by *aegopodiosa* vegetation type (Table 1). At the L10 site, *Larix* spp. grows in admixture with *Betula pendula* on the fertile soils of normal humidity characterized by *hepatico-oxalidosa* vegetation type (Table 1). Both *T. gabrieli* adults were trapped in the period between 10th of May and 5th of June and on trees that were 50–60 years old. At the L3 site, the tree was dead, while at the L10 site, the tree was living.

### 3.1. Morphology

**Figure 2:**
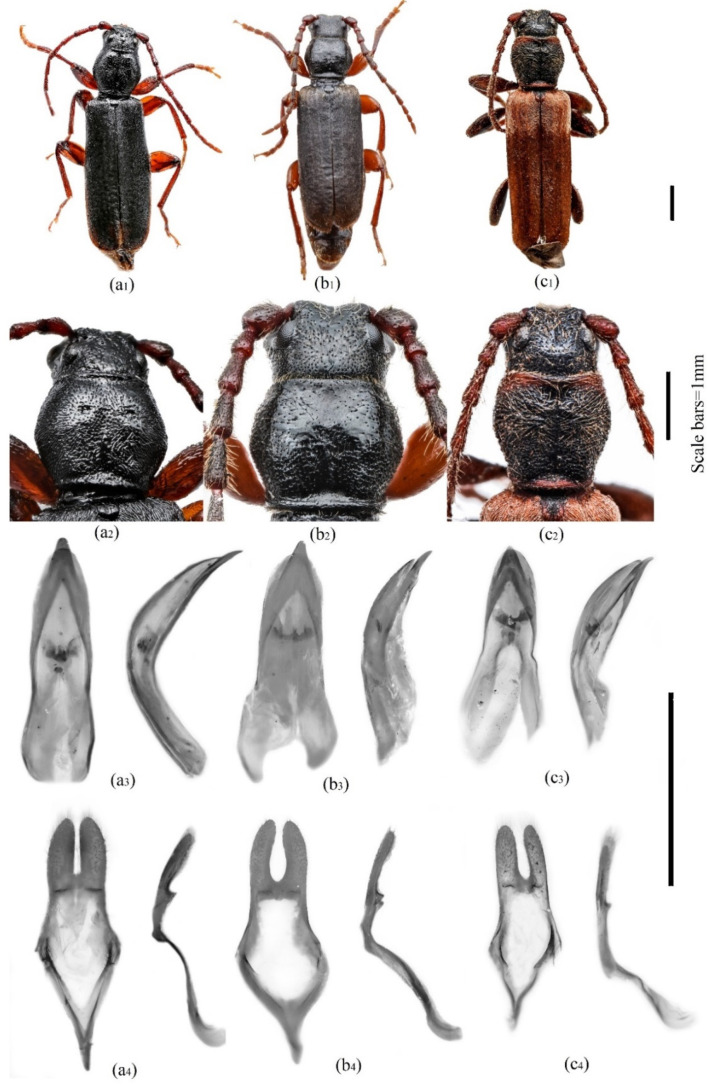
Comparative morphological characteristics among males of *T. gabrieli* (**a**), *T. castaneum* (**b**) and *T. fuscum* (**c**); (**a_1_**,**b_1_**,**c_1_**): habitus of adults (dorsal view); (**a_2_**,**b_2_**,**c_2_**): frontal part of habitus (dorsal view); (**a_3_**,**b_3_**,**c_3_**): adaeagus (ventral—in left side, and lateral in right side); (**a_4_**,**b_4_**,**c_4_**): parameres (ventral—in left side, and lateral in right side). The original photographs were prepared using the specimens collected in Lithuania and stored at T. Ivanauskas zoological museum, Kaunas, Lithuania.

The specimen trapped at the L10 site was used for the detailed morphological analysis. It was a male with a body length of 8 mm, dorsally and ventrally black and appendages being rufous (Figure 2). The front was flat, without longitudinal impression, pronotum shiny, disc densely and almost evenly punctured and elongated (length to width ratio was 0.892), and the sides were evenly convexed. Elytra are without distinct longitudinal edges and evenly rounded apically. Adaeagus was evenly pointed to the sharp apex, and parameres are wide with straight inner sides (Figure 2). The specimen studied presently possessed distinctive morphological features characteristic for *T. gabrieli* [13,23,26,27]. However, all three species are quite similar in body form (Figure 2) and differ in characters that must be examined using a microscope. Otherwise, subtle body proportions must be measured for species identity. In addition, we observed that the shape of pronotum (length to width ratio) and the shape of aedeagus could be good characters to distinguish *T. gabrieli* from *T. castaneum*, which also have a shiny disc of pronotum (Figure 2), which in some cases makes their separation quite problematic. As Sharp [28] shows, another character that distinguishes between these two species is that *T. castaneum* has a more strongly raised basal margin of its thorax than *T. gabrieli*.

An identification key for three *Tetropium* species is presented below:

1. The disc of the pronotum is matt, densely and rugosely punctured, pubescence with snuggled or hemi erected short hairs; a medial groove is distinct (Figure 2(c2)). A basal part of the elytra (about one-fourth of total elytra length) is covered by silver shine hairs (Figure 2(c1)). Inner sides of parameres are slightly curved; (Figure 2(c4))—*T. fuscum*.
  - The disc of the pronotum is shiny, densely, or sparsely punctured, pubescence with very short and closely snuggled hairs; the median groove is very shallow, indistinct (Figure 2(a2,b2)). Entire elytra are evenly coloured, covered by dark hairs. Inner sides of parameres are curved or straight.
2. The disc of the pronotum is densely and almost evenly punctured; its length to width ratio is about 0.9; forehead without longitudinal impression between eyes (Figure 2(a2)). The inner sides of parameres are straight; adaeagus is curved dorsally at least as for 65° (Figure 2(a3,a4))—*T. gabrieli*.
  - The disc of pronotum is sparsely and unevenly punctured with somewhere absolutely unpunctured areas in central part, its length to width ratio is about 0.8; forehead with a distinct groove between eyes (Figure 2(b2)); Inner side of parameres are distinctly curved; adaeagus is curved dorsally at least as for 40° (Figure 2(b3,b4))—*T. castaneum*.

### 3.2. Geographic Distribution

A few specimens of *Tetropium*, which were described by Weise, 1905 [23] as a *T. gabrieli* as a new species, were collected in the mountain regions of Helvetia (Switzerland), Tirol (Austria) and Silesia (Poland). Several specimens of *Tetropium* were collected in Great Britain, and these were described by Sharp (1905) [28] as a new species *T. crawshayi*. However, the latter two names were considered to be synonyms of the same species [29]. Possibly *T. gabrieli* was introduced to Great Britain in the 16th or 17th century, i.e., when cultivation of *Larix* spp. trees was beginning there [30]. *T. gabrieli* was also discovered in Germany, France, Denmark [26,31,32]. As a pest, *T. gabrieli* has received greater attention in the middle of the 20th century in Germany when areas with monocultures or mixed forest stands of *Larix* spp. were greatly expanded [13]. Then, *T. gabrieli* rapidly expanded to the north and since 1970, it has established in Poland, likely due to the increased planting of *Larix* spp. trees [33]. Despite the accidental entry of *T. gabrieli* to Sweden with imported timber wood in the 1990s [25], a broader establishment was only observed in 2007 [7]. In 1990, this species was also imported to Finland [34], but it appears to not have been established yet. In Europe, the distributional range of *T. gabrieli* expands from Ireland in the west to Belorussia and Ukraine in the east, and from Sweden (58°) in the north to southeastern France [35], Switzerland, Austria, and Hungary [1].

In the last decade, *T. gabrieli* was found close to the southern Lithuanian border, i.e., in the Jurkiszki area, Poland [36] and was recorded in the Białowieża forest from both Polish and Belorussian sides [33,37]. However, the direction *T. gabrieli* expanded into Lithuania remains unclear as no specimens were detected at the study sites in southern Lithuania (Figure 1). As several different *Larix* species were planted in Lithuania during the last 200 years [15], the possibility should not be excluded that *T. gabrieli* established here some time ago, persisting in small and scattered populations, thereby causing only limited damage to colonized trees. Moreover, xylophagous beetles associated with *Larix* spp. have not been studied before. For example, Duffy [38] has noted that in Great Britain, *T. gabrieli* usually attacks damaged or recently felled trees only.

### 3.3. Life Cycle

The life cycle of *T. gabrieli* was studied in detail during the last century [13,26,29,39]. The life cycle appears to be very similar to other *Tetropium* species, e.g., *T. castaneum* and *T. fuscum*. However, *T. gabrieli* was found to be nearly exclusively monophagous on *Larix* spp. trees [13,39,40]. Studies by Crawshay [29] and Schimitschek [26] have suggested that *Picea* and *Pinus* could also be suitable hosts for this longhorn beetle. Generally, the generation lasts one year, but Crawshay [29] and Duffy [38] have stated that high temperatures can lead to a shorter generation time and the emergence of the second generation in the same year. In Great Britain and Germany, adults normally appear in early June and last until the end of the first week in July [26,29,39]. Females lay up to 130 eggs between the scales of the outer bark. Interestingly, Crawshay [29] has found these beetles to be exclusively diurnal in their habitats, while Sláma [40] regarded this species as active at dusk or even nocturnal.

Several studies demonstrated that climate change and the increase in prolonged droughts were among the main predisposing factors for the following attacks by *T. gabrieli* on *Larix* spp. [8,41]. Thus, a similar situation can be expected in the near future in Lithuania. In this study, *T. gabrieli* beetles were accidentally captured using sticky traps, which are not typically used to investigate Cerambycid insects. Therefore, the results presented here should be interpreted with caution as these may not reflect the real distribution of *T. gabrieli* in *Larix* spp. stands in Lithuania. However, the first findings of *T. gabrieli* indicate the need for more detailed studies using more effective assessment methods. For example, such methods could include the evaluation of larvae in spring-felled larch trap wood [7] or the capture of adults in attractant-baited traps during the flying period [8]. In the future, such studies can be of potential practical importance in providing timely and valuable information, which may help understand the biology and ecology of *T. gabrieli* and sustain the health and the growth of *Larix* spp. stands.

## Figures and Tables

**Table 1:** Characteristics of investigated *Larix* spp. stands. Information is based on the forest inventory data obtained from the State Forest Cadastre as of 1 December 2020.

Site No. *	Geographical Position	Age (y)	Mean Height (m)	Mean Diameter (cm)	Stocking Level	Forest Site Type **	Forest Vegetation Type ***	Tree Species Composition (%) ****
**L1**	54°33′19.82″ N, 23°53′17.18″ E	47	28.1	34.3	0.9	Ncs	ox	100L
**L2**	54°51′37.18″ N, 24°4′29.02″ E	37	28.5	33.7	0.8	Ncp	ox	90L10T
**L3**	55°17′10.56″ N, 23°26′23.63″ E	50	29.1	43.5	0.7	Ldp	aeg	100L
**L4**	55°3′18.74″ N, 23°31′4.2″ E	72	35.9	42.8	0.8	Ncl	ox	90L 10P
**L5**	55°57′51.93″ N, 25°37′7.89″ E	80	28.0	34.0	0.6	Ldp	aeg	70L 20Pt 10B
**L6**	55°30′46.9″ N, 25°5′35.92″ E	55	25.0	24.0	0.9	Lcl	ox	50P 30L 20S
**L7**	55°15′53.53″ N, 24°48′50.76″ E	38	24.7	29.4	0.9	Ncl	ox	100L
**L8**	54°49′24.5″ N, 23°25′29.83″ E	66	32.7	32.2	0.8	Ncl	ox	80L 20P
**L9**	54°0′20.68″ N, 23°38′7.07″ E	59	32.2	38.6	0.6	Ncl	ox	100L
**L10**	55°23′14.38″ N, 24°7′13.74″ E	58	26.4	29.2	0.7	Nds	hox	90L 10B

* L1–L10: *Larix* spp. stands as in Figure 1. ** N: Normal humidity soils, L: temporarily waterlogged mineral soils, c: moderate fertility, d: high fertility, l: light soil texture, p: two-layered soil structure with a light fraction on a heavy fraction or vice versa, s: heavy soil texture; [19]. *** ox: *oxalidosa*, hox—*hepatico-oxalidosa*, aeg: *aegopodiosa* [20]. **** L: *Larix* sp., S: *Picea abies*, P: *Pinus sylvestris*, B: *Betula pendula*, T: *Tilia cordata*, Pt: *Populus tremula*, in each stand, tree species composition is based on the volume.

## Data Availability

The morphological data presented in this study are available on request from the corresponding author.
